# Mapping X-Disease Phytoplasma Resistance in *Prunus virginiana*

**DOI:** 10.3389/fpls.2017.02057

**Published:** 2017-11-29

**Authors:** Ryan R. Lenz, Wenhao Dai

**Affiliations:** Department of Plant Sciences, North Dakota State University, Fargo, ND, United States

**Keywords:** chokecherry, consensus map, tetraploid, QTL mapping, X-disease, phytoplasma, *Prunus*

## Abstract

Phytoplasmas such as “*Candidatus* Phytoplasma pruni,” the causal agent of X-disease of stone fruits, lack detailed biological analysis. This has limited the understanding of plant resistance mechanisms. Chokecherry (*Prunus virginiana* L.) is a promising model to be used for the plant-phytoplasma interaction due to its documented ability to resist X-disease infection. A consensus chokecherry genetic map “Cho” was developed with JoinMap 4.0 by joining two parental maps. The new map contains a complete set of 16 linkage groups, spanning a genetic distance of 2,172 cM with an average marker density of 3.97 cM. Three significant quantitative trait loci (QTL) associated with X-disease resistance were identified contributing to a total of 45.9% of the phenotypic variation. This updated genetic linkage map and the identified QTL will provide the framework needed to facilitate molecular genetics, genomics, breeding, and biotechnology research concerning X-disease in chokecherry and other *Prunus* species.

## Introduction

Fruit and tree nut production contributes about $25 billion to the U.S. economy annually (https://www.ers.usda.gov/topics/crops/fruit-tree-nuts/). Demand for fresh fruit is still growing; however, limiting factors such as plant disease reduce yield potential and plant survival in commercial production systems. X-disease, caused by *Candidatus* Phytoplasma pruni, is a major example which affects a variety of *Prunus* species such as peach, apricot, nectarine, cherry, plum, and chokecherry (Guo et al., 1998; Davis et al., 2013). Disease incidence as high as 60% and yield reductions ranging from 30 to 80% have been observed in Connecticut peach orchards (Douglas, 1999) and more importantly can cause more than 50% mortality 3 years post-infection (Peterson, 1984). Despite numerous investigations, phytoplasma biology and mechanisms of plant resistance is not well-understood. This is why disease management is often limited to complete eradication of infected trees (Chkhaidze et al., 2016). Other control measures for X-disease phytoplasma include pesticide treatment of leafhopper vectors, antibiotic treatment, non-host buffer zones, and disease resistant cultivars if available. Besides plant resistance, these methods are known to be inefficient and expensive (Peterson, 1984; Davis et al., 2013).

It is commonly argued that X-disease resistant cultivars offer the best method of phytoplasma control; however, natural resistance to X-disease hasn't been well-documented except for in chokecherry (Peterson, 1984; Guo et al., 1998; Davis et al., 2013; Wang et al., 2014). Chokecherry, a native tree species to North America and a natural host to X-disease phytoplasma, is the primary source of X-disease because it is a dominant reservoir of leafhoppers, by which the X-disease phytoplasma is vectored and transmitted. Those make chokecherry a potential model for genetic studies involving disease resistance and host-pathogen interactions of phytoplasma diseases. In 1983, a chokecherry seed source located in Bismarck, ND, was established by the United States Department of Agriculture-Natural Resources Conservation Service (USDA-NRCS) to examine potential X-disease resistant materials. Over 3,000 established germplasm from 179 seed sources collected from ND, MN and the surrounding region were planted and evaluated for X-disease symptoms. By 1994, 44% of the plants were dead and the remaining plants still contained X-disease phytoplasma. Only 5% of the remaining plants displayed little to no X-disease damage. Walla et al. (1996) reported that the few plants with little damage and/or zero observable symptoms might be resistant or highly tolerant to X-disease phytoplasma. This chokecherry planting paved the way for recent molecular genetic studies in chokecherry (Wang et al., 2014) and the results presented here.

Chokecherry has the same base chromosome number as other *Prunus* species (*x* = 8), but is one of the few tetraploids, having 32 chromosomes (2*n* = 4*x* = 32) (Dai, unpublished). Complex inheritance in tetraploids, particularly autotetraploids, makes genetic analysis of this species a challenge. Random combinations of bivalent pairing and quadrivalent pairing from four homologous chromosomes and genetic anomalies like double reduction are a few examples leading to the complexity (Mather, 1935; Gar et al., 2011). Cytological determination of the inheritance pattern (the chromosome pairing behavior) for tetraploid chokecherry is difficult due to its small chromosome size. Allotetraploids have similar inheritance as diploids and are easier to achieve accurate genetic mapping vs. autotetraploids; however, many tetraploid species have intermediate inheritance in which some chromosomes have diverged enough to preferentially pair, while others are similar enough to have levels of random pairing during meiosis (Hickok, 1978a,b; Stift et al., 2008; Koning-Boucoiran et al., 2012).

Understanding the inheritance mode of each chromosome can assist linkage analysis of molecular markers for the construction of reliable genetic linkage maps and QTL analysis. Unfortunately, the uncertainty of chokecherry inheritance hinders straightforward genetic mapping in this important species. Nevertheless, Wu et al. (1992) proposed to detect and estimate linkage in a population of polyploids using single dose restriction fragments (SDRF) to overcome the difficulty in genetic mapping based on the fact that a single dose allele produces simplex by nulliplex arrangements that have consistent estimation parameters for recombination frequencies. Genetic linkage mapping based on the “single dose allele” or “single dose marker” (SDM) strategy has been successfully applied in many tetraploids (Beaver and Iezzoni, 1993; Barcaccia et al., 2003; Canli, 2004; Koning-Boucoiran et al., 2012; Tsai, 2013). Software programs including TetraploidMap (Hackett and Luo, 2003; Hackett et al., 2007) and JoinMap (Van Ooijen, 2006) are typical applications for genetic mapping of plants and both can be used for tetraploids depending on the approach (Bradshaw et al., 2008; Hackett et al., 2013; Massa et al., 2015; McCallum et al., 2016). For example, autotetraploid blueberry has been successfully mapped using TetraploidMap and JoinMap. McCallum et al. (2016) utilized SNPs and SSR markers to construct the first representative linkage groups in blueberry. They used TetraploidMap first to identify the groups, and then JoinMap was used to refine the maps one linkage group at a time. Additionally, JoinMap has the advantage of being able to combine parental maps based on shared markers. This function was utilized resulting in an improved blueberry genetic map and a framework to conduct future studies like QTL mapping. This is similar to the approach taken in this study.

Previous work has developed a partial genetic linkage map for each parent (RC and SC) of a chokecherry mapping population (Wang et al., 2014). A total of 302 markers were assigned to 14 linkage groups of the RC map and 259 markers were assigned to 16 SC linkage groups, covering 2,089 and 1562.4 cM of the genome, respectively. The marker density was 6.9 cM for the RC and 6.0 cM for the SC map. One quantitative trait locus (QTL) associated with X-disease resistance was detected. The QTL accounted for 26% of the total phenotypic variation. Marker positions and intervals may be different in different linkage maps. Thus, a consensus map combining the map information from both parental maps can lead to a more accurate reference for marker and QTL positions. Previous maps have large gaps and unassigned linkage group segments, which warrants the improvement of the chokecherry linkage map. An improved linkage map will provide more useful genetic resources for QTL identification, map-based cloning, and facilitated germplasm enhancement through molecular breeding technology.

The objectives of this research were to develop and utilize an improved chokecherry genetic linkage map for identifying genetic regions related to X-disease resistance. Simple sequence repeats (SSRs), amplified fragment length polymorphism (AFLP), and long terminal repeats (LTRs) markers were utilized in improving the genetic linkage map of chokecherry. Following the development of improved linkage maps, marker-assisted breeding and map-based cloning for X-disease resistance can be explored straightaway. Providing new information and resources for elucidating mechanisms involved with X-disease and other phytoplasma-derived diseases will advance future research regarding disease response. The research presented will allow for continued exploration aimed at identifying specific genes associated with natural resistance mechanisms in chokecherry, which can be used as a template for examining resistance in other *Prunus* species.

## Materials and methods

### Mapping population and its evaluation

The mapping population used for construction of the first chokecherry map (“RC × SC”) was used again in this study (Wang et al., 2014). The mapping population consists of 101 progenies which derived from a cross between a resistant chokecherry parent (RC) as the female and a susceptible chokecherry parent (SC) as the male. The two parental lines were selected from a large chokecherry germplasm collection that was established in 1983 by the USDA Natural Resources Conservation Service (NRCS). Chokecherry hybrid seedlings were inoculated with an aggressive X-disease phytoplasma strain collected in Fargo, ND via a side grafting method (Wang et al., 2014). In brief, scions consisting of fresh symptomatic chokecherry branches were collected from the source tree <2 h before being grafted to the stem of the seedling. Non-inoculated seedlings were used as controls. After 2 weeks of growth, nested PCR was used to confirm X-disease infection. Nested PCR utilized both universal phytoplasma primers (R16 F2- ACGACTGCTGCTAAGACTGG and R16 R2-TGACGGGCGGTGTGTACAAACCCCG) and X-disease specific primers (R16 (III) F2-AAGAGTGGAAAAACTCCC and R16 (III) R1-TCCGAACTGAGATTGA). For more information on nested PCR conditions see Wang et al. (2014). Segregation of X-disease resistance in the mapping population was evaluated over 4 years based on phenotype scores ranging from zero to five: zero indicates plant death and five indicates completely resistant and healthy plants (Wang et al., 2014).

### SSR marker analysis

A total of 48 peach simple sequence repeat (SSR) marker primer pairs were designed (Supplementary Table 1). Previous study on identification of candidate genes associated with X-disease resistance in chokecherry through comparative genomics showed that scaffolds 2, 4, and 6 may have potential resistance (R) genes (Liang et al., 2014); therefore peach SSRs were searched from the three scaffolds of the peach reference genome (The International Peach Genome Initiative et al., 2013) using the online software “RepeatMasker” (Smit et al., 2013). Primers were designed based on the flanking regions of the SSRs using Primer Premier 5.0 (http://www.premierbiosoft.com). Additional SSR markers from recent publications were also adopted. A group of 176 peach SSR markers that amplified products in sweet cherry (Dettori et al., 2015) and 11 pear SSR markers that were reported transferable in rosaceous species (Zhang et al., 2014) were used in this study. The 235 SSR markers were tested in a subpopulation (*n* = 8) of chokecherry to identify polymorphic banding patterns. Polymerase chain reaction (PCR) was conducted according to the method of Liang et al. (2016); however, changes in annealing temperature were made according to the primer characteristics (Supplementary Table 2). New SSR primers that amplified polymorphic bands within the subpopulation and the 257 qualified SSR markers from Wang et al. (2014) were used to run the full mapping population.

### LTR marker analysis

Long terminal repeat (LTR) retrotransposon markers were developed from a partial genome sequence of chokecherry (Liang et al., 2016). A total of 78 LTR primers that showed polymorphism in the chokecherry subpopulation and an addition of eight highly polymorphic and multi-allelic pear LTR-based markers (Sun et al., 2015) were used to run the full mapping population. Information on chokecherry and pear LTR markers that were tested in the mapping population can be found in Liang et al. (2016) and Supplementary Table 3, respectively.

### AFLP markers

A total of 241 amplified fragment length polymorphism (AFLP) markers qualified during the previous research of tetraploid chokecherry mapping (Wang et al., 2014) were re-evaluated for the usability in this research.

### Map construction

Linkage analysis was performed using JoinMap 4.0 (Van Ooijen, 2006) and the cross-pollinating (CP) population type. A total of 1077 molecular markers were scored for the presence/absence of individual marker alleles. This method circumvents the mathematical differences between disomic and tetrasomic genetic linkage calculations as discussed before. Single dose alleles of all molecular markers were coded as absent or present in reference to the parental type (nn × np, lm × ll). Map constructions were performed following a “Two-Step” strategy that involved constructing parental maps separately before combining (Tavassolian et al., 2010; Klagges et al., 2013). The segregation pattern of markers was tested and distorted markers (determined by a chi-square threshold of 0.001) were eliminated from analysis. “Suspect Linkage” and “Genotype Probabilities” tabs were used to identify mis-grouped markers and double recombination, respectively. Regression mapping was used as the mapping algorithm with Kosambi's mapping function to convert recombination frequency into map distance. A minimum logarithm of odds (LOD) score established the linkage groups. Analyzing the strongest cross-link (SCL) parameter helped identify proper linkage group assignment of the markers. Ungrouped markers were manually transferred into established groups by examining SCL and related LOD values.

The process of removing unfit loci, reassigning groups, and mapping was done for each individual parental map until a limited number of markers could not be assigned to a linkage group. Final linkage groups were compared between each parental map to define a consensus grouping based on homologous loci. Every linkage group was separately aligned with each consensus parental grouping to check for conflicting markers before finalizing consensus groups. Merged chokecherry linkage groups were developed with the “Combine Groups for Map Integration” function of JoinMap; however, MergeMap Online (Wu et al., 2008) was used to finish combining linkage groups that did not successfully order in JoinMap. All combined chokecherry linkage groups were drawn using MapChart 2.30 (Voorrips, 2002) and compared with the previous chokecherry “RC × SC” map (Wang et al., 2014) (Supplementary Figure 1).

### QTL analysis

The merged linkage groups derived from the parental chokecherry maps were used for QTL analysis via QGene 4.3.10 (Joehanes and Nelson, 2008). Normality test of the phenotypic data was reported as a Kolmogorov-Smirnov (K-S) *p*-value. Composite Interval Mapping (CIM) was used to detect quantitative trait loci (QTL). Nearby loci with the highest LOD scores were selected as cofactors per the default parameters set in the program. Permutation tests with 1,000 iterations were used to determine significant LOD thresholds at the 95% and 99% confidence levels for the experiment-wise Type I error.

Identified QTL and the nearest markers were further analyzed to discover candidate genes linked to the region. This method utilizes ideas from ePCR and sequence alignment to extract DNA sequences located within QTL-flanking markers (Schuler, 1997; Sivasubramanian et al., 2017). In general, the Genome Database for Rosaceae (Jung et al., 2014) was used to align the markers to the peach (*Prunus persica*) and the sweet cherry (*Prunus avium*) genomes. The Genome Database for Rosaceae contains the most updated genomes for Prunus (Shirasawa et al., 2017; Verde et al., 2017), so that is why it was used instead of just NCBI's BLAST server. DNA sequences between QTL-flanking markers were subsequently aligned via BLASTn to identify homologs with relevant gene annotations. The gene annotations were screened on the UniProtKB website (The UniProt Consortium, 2017) for gene ontologies related to disease resistance, biotic stress, and cell regulation proteins (e.g., transcription factors).

### Comparative analysis of *Prunus* genetic maps

Synteny analysis with other *Prunus* maps was conducted. The linkage groups from the consensus chokecherry map “Cho” (linkage groups 1 to 16) were compared to the *Prunus* reference map (“T × E”) (Joobeur et al., 1998; Aranzana et al., 2003; Dirlewanger et al., 2004), the sweet cherry linkage map (“EF × NY”) (Olmstead et al., 2008), and the peach reference genome (The International Peach Genome Initiative et al., 2013; Dettori et al., 2015). Homologous loci between the new chokecherry map (“Cho”) and the “T × E,” “EF × NY” maps, and the peach psuedochromosomes are reported. The “Cho” map produced herein was also compared to the previous chokecherry map “RC × SC” (Wang et al., 2014). MapChart 2.30 was used to visualize how the new map combined the parental maps and the small chromosome segments (data not shown).

A flowchart that describes the steps taken to create a genetic linkage map and identify significant QTL in this study was provided in Supplementary Figure 2.

## Results

### Molecular markers and chokecherry genetic mapping

A total of 176 peach and 11 pear SSR primers previously published were tested for their transferability in a chokecherry subpopulation (*n* = 8). Results showed that peach SSRs were more transferable in which 130 primers (73.9%) produced amplicons, while four pear SSRs (36.4%) were amplifiable in chokecherry (Table 1). Of 187 primer pairs, 117 (116 from peach and one from pear) showed polymorphisms and were used to run the full mapping population. There were 27 of the 48 newly developed peach SSR primer pairs that produced amplicons-−19 produced polymorphic markers (Table 1). Technical details for the 19 polymorphic peach SSRs are highlighted and summarized in Supplementary Table 1. Lastly, 78 polymorphic chokecherry LTR markers (Liang et al., 2016) were used in the full mapping population.

**Table 1:** Origin and overall performance of molecular markers tested in genetic mapping of the new chokecherry map “Cho”.

**Marker type**	**Source species**	**Tested**	**Amplified**	**Polymorphic**	**Qualified^c^**	**Mapped**	**Mapped^d^(%)**	**References**
SSR	Peach	176	130	116	85	54	30.7	Dettori et al., 2015
	Peach	48	27	19	17	11	22.9	Present study
	Pear	11	4	1	1	1	9.1	Zhang et al., 2014
LTR	Chokecherry	336	283	78	59	20	6.0	Liang et al., 2016
	Pear	8	0	0	0	0	0.0	Sun et al., 2015
SSR	*Prunus*^a^	257	257	257	257	251	97.7	Wang et al., 2014
AFLP	Chokecherry^b^	241	241	241	241	228	94.6	Wang et al., 2014
Total		1077	942	712	660	565	52.5	

a *Prunus SSR markers originate from chokecherry, peach, sweet cherry, wild cherry, Japanese plum, apricot, almond, and sour cherry*.

b *Chokecherry AFLP markers are described in more detail in Wang et al. (2014)*.

c *Markers were considered qualified if segregation distortion ratios did not exceed χ's test at (p < 0.001)*.

d *Percentage of markers that were mapped of the total markers tested*.

All polymorphic markers were subject to segregation analysis in the full mapping population. Markers were considered qualified if segregation distortion from 1:1 or 3:1 ratios did not exceed Chi-Square's test at (*p* < 0.001). A total of 66 SSR and 20 LTR markers were qualified and successfully anchored to the chokecherry genetic map (Table 1 and Supplementary Figure 1). Details on the distribution of all anchored markers are summarized in Table 2.

**Table 2:** Marker distribution and map statistics for the new chokecherry genetic map “Cho”.

**Linkage group**	**SSR**	**AFLP**	**LTR**	**TOTAL**	**Length (cM)**	**Average distance (cM)^a^**	**Longest gap (cM)^b^**
Cho-1	12	7	1	20	129.7	6.5	19.4
Cho-2	20	12	0	32	127.8	4.0	18.3
Cho-3	17	13	1	31	149.2	4.8	16.6
Cho-4	38	9	0	47	142.6	3.0	9.9
Cho-5	32	3	0	35	142.5	4.1	11.7
Cho-6	20	9	2	31	98.3	3.2	8.7
Cho-7	18	21	0	39	126.9	3.3	13.1
Cho-8	10	25	0	35	145.6	4.2	21.2
Cho-9	7	23	0	30	156.8	5.2	13.2
Cho-10	20	23	0	43	161.6	3.8	11.3
Cho-11	17	0	15	32	98.2	3.1	10.7
Cho-12	18	15	0	33	168.6	5.1	15.2
Cho-13	25	14	0	39	171.4	4.4	10.1
Cho-14	15	21	1	37	135.0	3.6	16.5
Cho-15	25	20	0	45	124.4	2.8	9.4
Cho-16	23	13	0	36	93.6	2.6	9.1
Total	317	228	20	565	2172.1	3.97	–

a *Average distance in centi-Morgans (cM) between markers per linkage group*.

b *Largest gap between markers per linkage group*.

A total of 257 SSR markers used previously in the first chokecherry map “RC × SC” were re-analyzed in this study. The scoring data by Wang et al. (2014) was converted to the JoinMap format and subjected to the same statistical tests described previously. The results showed that the majority (97.7%) of these SSR markers were successfully anchored to the new chokecherry linkage groups (Table 1). A total of 241 AFLP markers that were used in Wang et al. (2014) were also analyzed for their suitability in constructing the new chokecherry map and QTL mapping in this study. Of those, 228 (94.6%) were qualified and successfully mapped to the new chokecherry genetic map (Table 1).

### Map construction

Two JoinMap-generated parental genetic maps were created and analyzed to find homologous loci shared between parental linkage groups. Before combining two homologous groups, they were checked to make sure no shared loci in any other groups were found. Joining and re-ordering parental groups via the “Combine Groups for Map Integration” function of JoinMap successfully created a total of 12 new linkage groups. Four pairs of linkage groups did not order successfully even though they had shared loci. MergeMap Online was able to overcome this issue, and the last four linkage groups were created to complete the full genetic map representing the haploid chromosome number of chokecherry (*n* = 2*x* = 16) (Supplementary Figure 1).

The new chokecherry consensus linkage map (“Cho”) has a total genetic length of 2172.1 cM. The linkage group with the longest map distance was Cho-13, spanning 171.4 cM, whereas Cho-16 was the shortest at 93.6 cM (Table 2). The linkage group with the most markers was Cho-4, having 47 anchored markers consisting of 38 SSRs and nine AFLPs spanning a total map length of 142.6 cM. Linkage group 1 (Cho-1) anchored the fewest markers with 12 SSR, seven AFLP, and one LTR being distributed along 129.7 cM. The biggest gap between two adjacent markers was 21.2 cM near the end of Cho-8. Overall the marker density for all linkage groups was 3.97 cM (Table 2).

### Comparative analysis of the consensus map

The newly developed consensus linkage map “Cho” was compared to the previously published chokecherry “RC × SC” map (Wang et al., 2014). The new “Cho” linkage groups are numbered according to the homologous relationship to those of the “RC × SC” map. Most new linkage groups corresponded uniquely to previous linkage group pairs, but a few groups integrated different combinations of previous linkage group segments or none at all. For examples, linkage group Cho-1 in the “Cho” map corresponded best with SC-1 and RC-11 in the “RC × SC” map (data not shown) and Cho-2 corresponded best with RC-2 and SC-10. Cho-10 was mostly a combination of RC-10 and the segmented group SC-2b. Lastly, Cho-4 resembled the segmented groups RC-12a and SC-4e. These new combinations of linkage groups eliminated previous linkage groups that were labeled 12 and 11. To compensate, the two new chokecherry groups with the least resemblance to previous linkage groups were assigned as Cho-11 and Cho-12. Group Cho-11 showed zero homology with previous maps, while Cho-12 showed homology to segment 14b of the RC linkage map and the top half of SC-5. Overall, the length of the consensus map “Cho” was 2172.1 cM compared to the 2089.0 cM and 1562.0 cM for the previous RC and SC maps, respectively (Table 3), representing a 104% and 139% increase in genetic length for the new chokecherry map. The marker density was substantially increased, going from 6.9 cM (RC) and 6.0 cM (SC) in the “RC × SC” map to 3.97 cM per marker in the “Cho” map.

**Table 3:** Size comparison in centi-Morgans (cM) of the new chokecherry map “Cho” to reference maps in *Prunus*.

	**RC^a^**	**SC^b^**	**“T × E”^c^**	**“EF × NY”^d^**
Cho map	2172.1	2172.1	2172.1	2172.1
Reference map	2089.0	1562.0	621.2	638.5
Difference	83.05	610.1	1550.9	1533.6
Percent difference (%)	104	139	350	340

a *Resistant chokecherry (RC) parent map (Wang et al., 2014)*.

b *Susceptible chokecherry (SC) parent map (Wang et al., 2014)*.

c *“Texas” almond × “Earlygold” peach (“T × E”) reference map for Prunus species (Joobeur et al., 1998; Aranzana et al., 2003; Dirlewanger et al., 2004)*.

d *“Emperor Francis” × “New York 54” (“EF × NY”) sweet cherry map (Olmstead et al., 2008)*.

Syntenic relationships between the “Cho” map and the *Prunus* “T × E” map (Joobeur et al., 1998; Aranzana et al., 2003; Dirlewanger et al., 2004), the sweet cherry “EF × NY” map (Olmstead et al., 2008), and the psuedochromosomes of the peach reference genome (The International Peach Genome Initiative et al., 2013; Dettori et al., 2015) were examined (Table 4). It was discovered that 36 total loci are orthologous between the “Cho” map and the “T × E” map, 25 loci were shared with sweet cherry, and 90 loci were aligned to the peach psuedochromosome position (Table 5). The largest number of orthologous loci is seen with both Cho-4 and Cho-6 having eight orthologous loci with reference groups four and six, respectively (Table 4). Other strong relationships include Cho-2, Cho-7, and Cho-15 which have six orthologous loci found in their respective reference groups 7, 3, and 1 (Table 4). Cho-11 was the most diverse, having at least two loci from reference groups 4, 6, and 8.

**Table 4:** Number of shared markers for chokecherry and reference *Prunus* linkage groups: (“T × E”), (“EF × NY”), and psuedochromosomes^c^.

	**1^a^**	**2**	**3**	**4**	**5**	**6**	**7**	**8**
Cho-1^b^					2^c^			
Cho-2							6	
Cho-3	6	1		2				
Cho-4	1	1		8				
Cho-5				1	2	1		
Cho-6						8		
Cho-7			6					
Cho-8	2							
Cho-9			1		1	1		
Cho-10		3			2	1		1
Cho-11	1		1	2		4	1	3
Cho-12				4				
Cho-13		1			1			5
Cho-14		3		1				
Cho-15	6			2				
Cho-16						2	1	

a *Reference linkage group from “Texas” almond × “Earlygold” peach (“T × E” map), “Emperor Francis” × “New York 54” (“EF × NY” sweet cherry map), and psuedochromosome number from the peach reference genome (The International Peach Genome Initiative et al., 2013)*.

b *Chokecherry (“Cho”) linkage groups*.

c *Number of markers shared between linkage groups*.

**Table 5:** List of homologous loci and their corresponding linkage groups.

**Marker**		**Linkage group**			
**Name**	**Type**	**Cho^a^**	**T × E^b^**	**EF × NY^c^**	**Peach chrom^d^**
EMPaS11	SSR	1	–	5	5
CPSCT006	SSR	1	5	–	5
RPPG7-032	SSR	2	–	–	7
RPPG7-026	SSR	2	–	–	7
PMS2	SSR	2	7	7	7
RPPG7-023	SSR	2	–	–	7
CPSCT004	SSR	2	7	–	7
RPPG7-018	SSR	2	–	–	7
RPPG1-026	SSR	3	–	–	1
SSR8-E34	SSR	3	–	–	4
RPPG1-029	SSR	3	–	–	1
UDP97-402	SSR	3	2	–	2
RPPG1-041	SSR	3	–	–	1
PMS67	SSR	3	–	1	1
BPPCT027	SSR	3	1	–	1
BPPCT016	SSR	3	1	–	1
BPPCT036	SSR	3	4	–	1
UDP97-402	SSR	4	2	–	4
UDP98-024	SSR	4	4	–	4
PMS3	SSR	4	–	4	4
SSR8-D78	SSR	4	–	–	4
UDP98-022	SSR	4	–	1	1
SSR8-D56	SSR	4	–	–	4
RPPG4-074	SSR	4	–	–	4
BPPCT040	SSR	4	–	4	4
SSR8-E78	SSR	4	–	–	4
BPPCT014	SSR	5	5	5	5
BPPCT005	SSR	5	–	4	4
BPPCT032	SSR	5	5	–	5
RPPG6-018	SSR	5	–	–	6
EMPaS01	SSR	6	–	6	6
BPPCT008	SSR	6	6	6	6
RPPG6-030	SSR	6	–	–	6
RPPG6-010	SSR	6	–	–	6
UDP98-412	SSR	6	6	–	6
RPPG6-018	SSR	6	–	–	6
RPPG6-024	SSR	6	–	–	6
CPSCT012	SSR	6	6	–	6
RPPG3-031	SSR	7	–	–	3
RPPG3-030	SSR	7	–	–	3
RPPG3-039	SSR	7	–	–	3
PMS30	SSR	7	–	3	3
BPPCT039	SSR	7	3	–	3
BPPCT007	SSR	7	3	–	3
PMS67	SSR	8	1	1	1
BPPCT028	SSR	8	1	–	1
BPPCT026	SSR	9	5	5	5
BPPCT009	SSR	9	6	6	6
CPDCT008	SSR	9	3	–	3
BPPCT002	SSR	10	2	2	2
BPPCT006	SSR	10	8	2,6	8
BPPCT001	SSR	10	2	–	2
BPPCT017	SSR	10	5	–	5
RPPG5-008	SSR	10	–	–	5
RPPG4-076	SSR	11	–	–	4
RPPG7-029	SSR	11	–	–	7
RPPG8-020	SSR	11	–	–	8
RPPG6-038	SSR	11	–	–	6
RPPG6-036	SSR	11	–	–	6
RPPG1-025	SSR	11	–	–	1
RPPG6-014	SSR	11	–	–	6
RPPG4-097	SSR	11	–	–	4
RPPG8-017	SSR	11	–	–	8
RPPG8-031	SSR	11	–	–	8
RPPG3-041	SSR	11	–	–	3
RPPG6-009	SSR	11	–	–	6
PMS3	SSR	12	4	4	4
SSR8-F78	SSR	12	–	–	4
BPPCT040	SSR	12	4	4	4
RPPG4-084	SSR	12	–	–	4
CPSCT021	SSR	13	2	2	2
BPPCT012	SSR	13	8	–	8
RPPG8-014	SSR	13	–	–	8
RPPG8-011	SSR	13	–	–	8
RPPG8-007	SSR	13	–	–	8
BPPCT032	SSR	13	5	–	5
RPPG8-030	SSR	13	–	–	8
BPPCT013	SSR	14	2	4	2
BPPCT002	SSR	14	2	2	2
RPPG2-019	SSR	14	–	–	2
SSR8-D1112	SSR	15	–	–	4
RPPG1-017	SSR	15	–	–	1
RPPG1-023	SSR	15	–	–	1
PceGA59	SSR	15	1	1	1
UCD-CH31	SSR	15	–	1	1
BPPCT027	SSR	15	1	–	1
BPPCT036	SSR	15	4	–	1
RPPG6-033	SSR	16	–	–	6
UDP98-021	SSR	16	–	6	6
UDP98-408	SSR	16	7	–	7
Total homologous loci shared:			36	25	90

a *Chokecherry (“Cho”) linkage groups*.

b *“Texas” almond × “Earlygold” peach (“T × E”) linkage groups*.

c *“Emperor Francis” × “New York 54” (“EF × NY”) sweet cherry linkage groups*.

d *Psuedochromosome number from the peach reference genome (The International Peach Genome Initiative et al., 2013)*.

### QTL mapping for X-disease resistance

**Figure 1:**
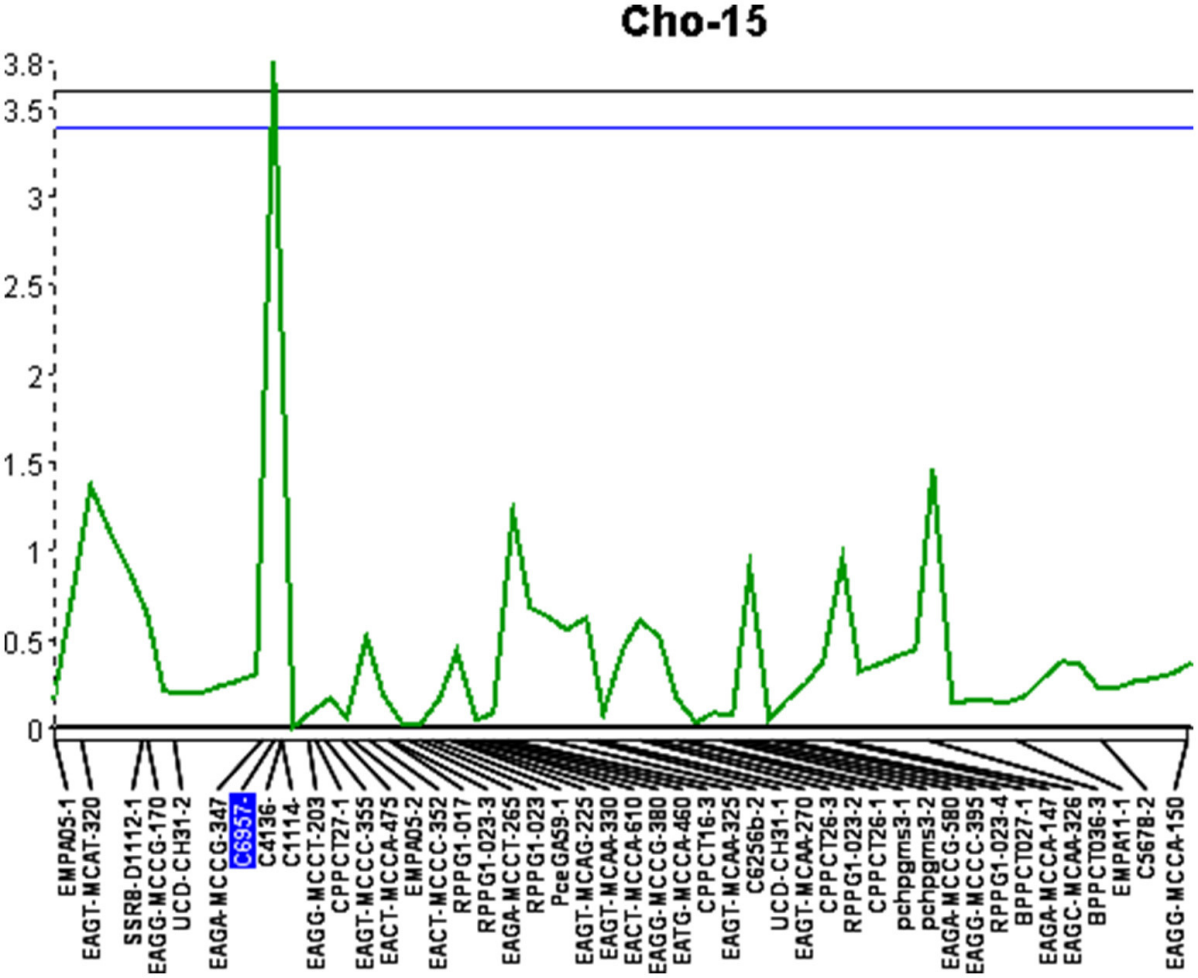
Quantitative trait locus identified on linkage group 15 (Cho-15) with an LOD score of 3.8. Upper and lower lines represent 1 and 5% significance thresholds, respectively.

QGene 4.0 was used for QTL identification in the consensus chokecherry map “Cho.” Phenotypic data of X-disease resistance in the mapping population was adopted from the previous research (Wang et al., 2014). Three significant QTL were identified (Table 6). The percentage of phenotypic variation explained (*R*^2^) was estimated for all three QTL as well as the mean genotypic values for each locus (Table 7). A QTL accounting for the greatest contribution of X-disease phenotypic variation was identified on linkage group Cho-15 (Figure 1). This particular locus spanned a distance of 2.1 cM, accounted for 18.4% of the phenotypic variation, had an additive effect of 0.71 on the phenotype score, and was significant at 99% levels of confidence (Table 6). The flanking AFLP marker, EAGA-MCCG-347, is linked to resistance at QTL-1 on Cho-15 if it has a single band allele which is the same genotype of the resistant parent (Table 7). If this marker band/allele is absent, then it shares the same genotype with the susceptible parent at that locus. The SSR marker nearest to the QTL on Cho-15 (C4136) gives a single band which is associated with the susceptible parent. In other words, increased resistance is associated the absence of a PCR amplicon from C4136 (Table 7).

**Table 6:** Significant quantitative trait loci (QTL) statistics and associated marker distances in centi-Morgans (cM).

**QTL**	**Linkage group**	**Position (cM)**	**Peak (LOD)**	**PS^a^**	**Additive effect^b^**	**Phenotypic variance^c^**	**Flanking marker 1**	**Flanking marker 2**	**Interval (cM)^d^**
1	Cho-15	24	3.8	^**^	0.71	18.4%	EAGA-MCCG-347	C4136	2.1
2	Cho-5	138	3.0	^**^	0.42	14.6%	C3637	C1795	11.5
3	Cho-4	78	2.6	^*^	0.66	12.9%	EAGT-MCCT-273	UDP98-024-3	6.9

a Permutation significance; Significance thresholds were set after 1,000 permutation iterations:

* Significant at α = 0.05 and

** *Significant at α = 0.01*.

b *Additive effect represents the phenotypic score change due to QTL genotypes matching the resistant parent*.

c *Phenotypic variance represents the R^2^-value produced by the QTL*.

d *Interval is the genetic distance between the flanking markers in which the QTL resides*.

**Table 7:** Summary of genotypic values for markers flanking the identified QTL linked to X-disease resistance.

**QTL**	**Locus**	**Phenotypic mean^a^**	**Genotypic values of progeny^b^**		**Statistically significant^c^**
			**Present**	**Absent**	
Cho-15	EAGA-MCCG-347	3.00	3.13	2.87	No
Cho-15	C4136	3.00	2.48	3.56	Yes
Cho-5	C3637-	3.00	3.12	2.39	Yes
Cho-5	C1795-	3.00	3.19	2.06	Yes
Cho-4	EAGT-MCCT-273	3.00	3.25	2.40	Yes
Cho-4	UDP98-024-3	3.00	3.11	2.66	No

a *The phenotypic mean is the total mean of all 101 progeny phenotypes from a disease resistance scale (0–5), 5 being completely resistant*.

b *Genotypic values are the average disease resistance score of progeny having a single dose allele present or absent at the locus*.

c *Significant differences between locus genotypes were tested with the Kruskal–Wallis test (α = 0.05)*.

**Figure 2:**
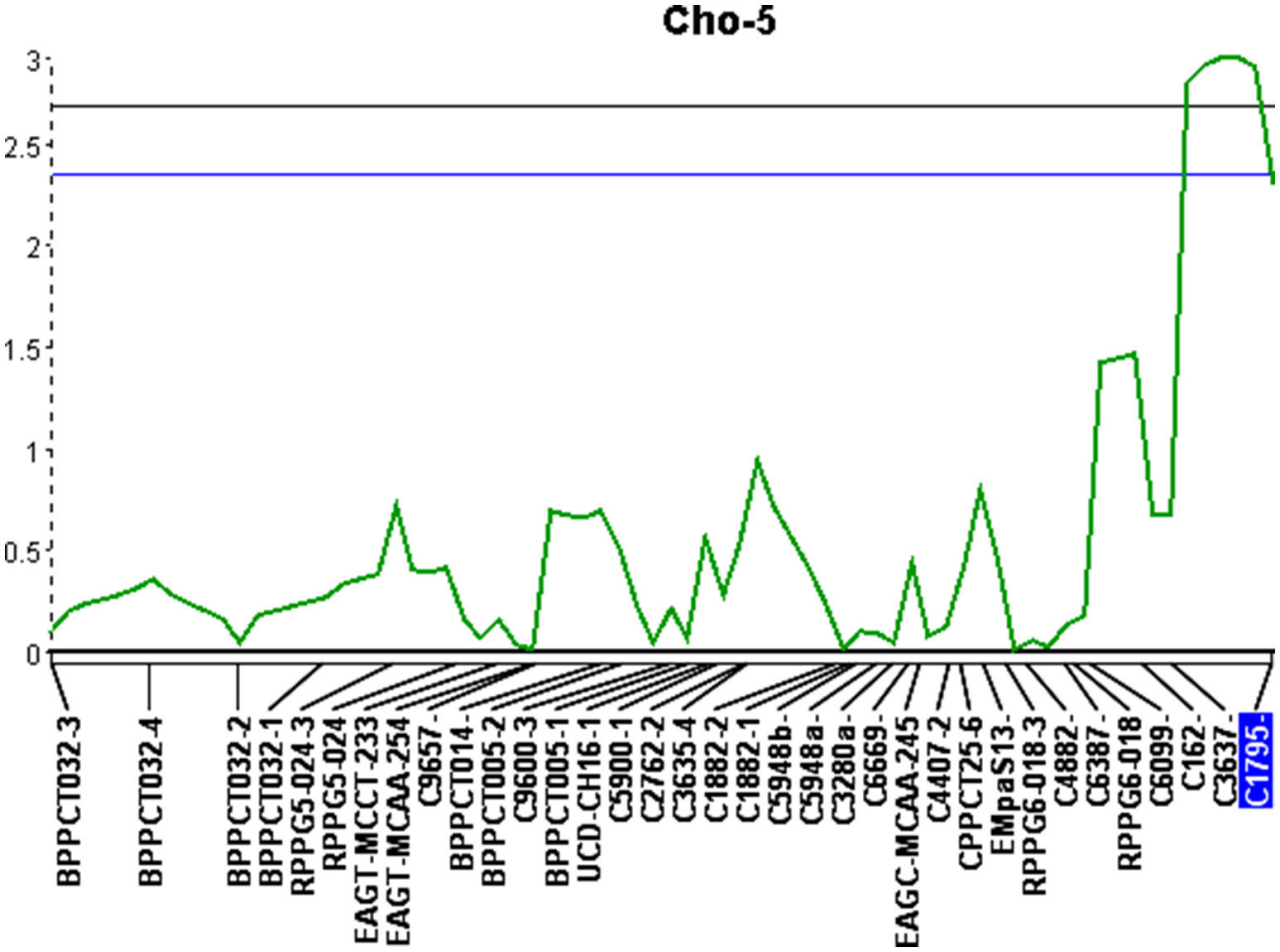
Quantitative trait locus identified on linkage group 5 (Cho-5) with an LOD score of 3.0. Upper and lower lines represent 1 and 5% significance thresholds, respectively.

The second significant QTL was located on linkage group Cho-5 (Figure 2). This particular locus explained 14.6% of the phenotypic variation, had an additive effect of 0.42, was significant at the 99% confidence level and spanned a genetic distance of 11.5 cM. The QTL located on Cho-5 is flanked by two chokecherry SSR markers, C3637 and C1795. They both give a single banding pattern. In both cases, resistance is linked to genotypes having the presence of a PCR band (Table 7).

**Figure 3:**
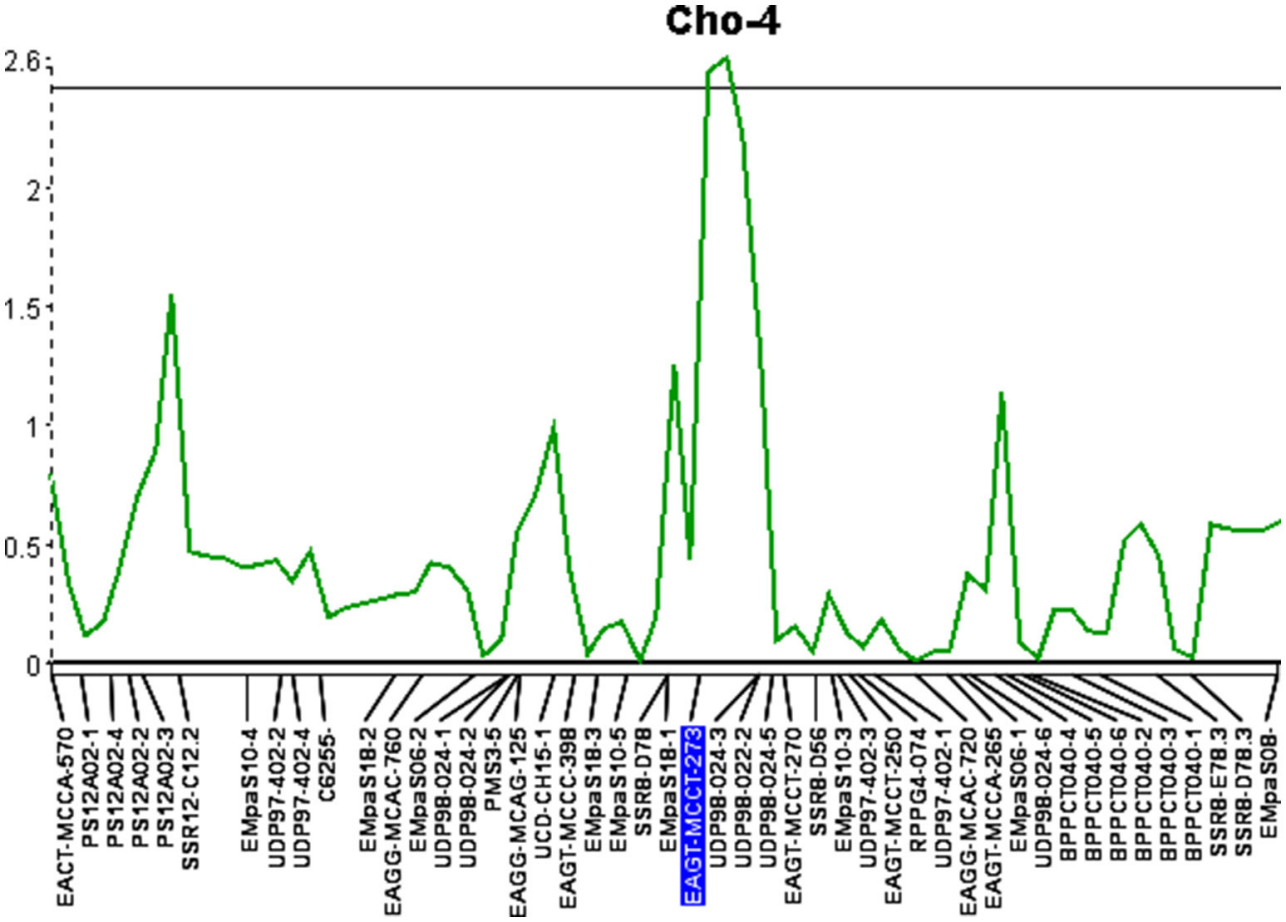
Quantitative trait locus identified on linkage group 4 (Cho-4) with an LOD score of 2.6. The horizontal line represents a significance threshold of 5%.

The third QTL was detected on Cho-4, accounted for 12.9% of the phenotypic variation and an additive effect of 0.66. This locus was significant at the 95% confidence level and spanned a distance of 6.9 cM (Figure 3). It is flanked by an AFLP marker and an SSR marker. The presence of AFLP marker, EAGT-MCCT-273, is linked to higher resistance (Table 7). The SSR marker flanking the QTL on Cho-4 produces a banding pattern of six amplicons in chokecherry. The presence of the third band/amplicon (middle band) is linked to higher resistance on average, but is not statistically different (Table 7).

All identified loci linked to X-disease resistance helped find a total of 70 disease related candidate genes from peach and 87 from sweet cherry (Supplementary Tables 4, 5). The majority of the gene ontologies correspond to disease resistance proteins such as cell membrane receptor kinases and signaling proteins including 31 putative NB-LRRs (Jones and Dangl, 2006). Other genes of interest include transcription factors, hormone signaling (e.g., abscisic acid and ethylene), antimicrobial biosynthesis (e.g., phytoalexin production), and structure-related proteins (Supplementary Tables 4, 5).

## Discussion

### Genetic mapping in understudied tetraploids

In an ideal situation, the inheritance pattern of each chromosome of chokecherry (2*n* = 4*x* = 32) would be established to conduct the most informative mapping method. For example, if it were found that chokecherry is primarily an allotetraploid with disomic inheritance, 16 linkage groups would remain as the most representative haploid number. If it were primarily an autotetraploid, then the 8 base chromosomes would need to represent the genetic map. Intermediate inheritance can complicate the linkage group number even further. Although the inheritance mode does not affect genetic maps created via the single-dose allele strategy, knowing it could increase the accuracy of mapping at an individual chromosome basis. This would increase the association reliability of the molecular markers, phenotypic traits, and chromosome position for marker-assisted selection and map-based cloning of important genes related to X-disease defense. Nevertheless, this research presents a simplified platform for developing necessary genetic tools in an understudied tetraploid plant species.

### Integration of chokecherry parental maps

Genetic maps produced from reliable molecular markers and heterogeneous populations are the basis for forward genetics, comparative genomics, and QTL identification. Chokecherry is the only plant species that is documented for X-disease resistance; however, very limited genetic resources are available. Wang et al. (2014) developed the first chokecherry genetic linkage map “RC × SC” consisting of individual parental linkage groups using TetraploidMap. Due to limited number of molecular markers and separate maps for both parents, the “RC × SC” map contained many large gaps (>30 cM) that resulted in 20 segments. A consensus map that combines parental maps can increases marker density and fill sizable gaps; ultimately, this increases fidelity and precision for QTL analysis due to the decreased interval between flanking markers and because the complete parental allelic contributions are considered (Keyser et al., 2010; Klagges et al., 2013; Wu et al., 2014).

In this study, Regression mapping in JoinMap was first used for each parental map separately. Regression mapping permits the construction of linkage groups by adding loci one at a time starting from the most informative pair of loci that were searched by comparing the goodness-of-fit of the calculated map for each tested position. After linkage groups for each parent was established, joining homologous groups was successfully implemented. A total of 12 pairs of parental linkage groups were successfully joined together. MergeMap was then used to join the remaining four groups. MergeMap relies on graph theory (Yap et al., 2003; Jackson et al., 2005) and uses directed acyclic graphs (DAGs) to represent maps from individual populations and to resolve conflicts between maps. MergeMap does not make use of genotype data, but simulations have shown that MergeMap can produce comparatively similar results in terms of accurately ordering molecular markers (Wu et al., 2008; Wang et al., 2011). It is important to understand that MergeMap relies solely on the linear arrangement of molecular markers from each paired parental map and does not use the genotypic data to perform the map re-calculation as in JoinMap. However, JoinMap has limited utility when a low number of shared markers are found between individual maps or a low frequency of genetic linkage is found for a connecting marker. Therefore, it is crucial that the original parental maps are a reliable representation of marker order and genetic distance especially when using MergeMap. Indeed, JoinMap and MergeMap can generate integrated maps with good consistency, so both have been used to construct the updated chokecherry linkage groups. Furthermore, the increase in marker density produced by combining parental maps have improved QTL mapping and have provided a resource for examination of genetic and physical positions (e.g., candidate gene identification, map-based cloning, comparative genomics, and genome sequencing).

### Syntenic relationship of chokecherry and other *Prunus* maps

Synteny is the product of shared chromosomal segments with the same genetic order between closely related species (Dirlewanger et al., 2004; Tang et al., 2008; Cabrera et al., 2009). Transferable markers can provide a means of determining synteny between two species. The reliability of the peach genome sequence and genetic maps have been utilized in studies to confirm synteny and collinearity of peach and *Prunus* species (Shulaev et al., 2008; Zhebentyayeva et al., 2008; Arús et al., 2012; Klagges et al., 2013). In this study, we conducted a syntenic analysis between the new chokecherry linkage map “Cho” and the *Prunus* reference map “T × E,” the sweet cherry genetic map “EF × NY,” and the peach reference genome. In spite of limited shared markers, linkage groups in the “Cho” map still show homology to representative chromosomes of *Prunus* (Tables 4, 5). However, chokecherry is a tetraploid and ancestral chromosome rearrangements and duplicated loci may have resulted in non-collinearity to other *Prunus* species. The relatively low number of shared markers between the “Cho” map and other *Prunus* maps may be illustrating this. Clearly, more shared markers and/or genome sequencing will help deduce the evolutionary relationship of chokecherry and *Prunus* species.

### QTL mapping for X-disease resistance in chokecherry

The success of QTL applications, such as marker-assisted selection and map-based cloning, largely depends on the reliability and accuracy of the QTL analysis and the underlying genetic linkage maps being used. In this study, a consensus linkage map of chokecherry was developed with the aim to identify additional QTL located near molecular markers. This updated map provides a solid framework for future studies to begin. Increased marker density and parental map integration, facilitated the discovery of three significant QTL associated with X-disease. The QTL located on linkage group Cho-15 contributed the most to the overall phenotypic effect and also had the shortest genetic interval of 2.1 cM. This particular QTL was previously identified with a 10-fold longer interval of 21.4 cM (Wang et al., 2014). Interestingly, the increased marker density and shorter QTL interval were associated with smaller phenotypic variation (18.4%) than previously reported (26.6%) at this locus. This may be attributed to the discovery of two additional QTL that explained 27.5% of the total phenotypic variation. Overall, the loci explained 45.9% of the phenotypic variation and had a cumulative additive effect of 1.79 to the phenotype scores (Table 6). Most QTL-flanking markers reported are in phase with the resistant parent and their presence increases the average resistance score by 1.79 in the 0–5 phenotype scale. Marker C4136 is the exception and flanks the QTL on Cho-15. It is in phase with the susceptible parent, therefore its presence may reduce the average phenotype score (Table 7).

Mapping QTL for X-disease resistance in chokecherry has helped reveal a collection of candidate genes based on BLAST hits and filtered gene ontology. Preliminary analysis of the orthologous QTL locations in sweet cherry and peach have provided a list of candidate genes within physical locations of flanking markers (Supplementary Tables 4, 5). Similar approaches have been used to link genetic markers to physical locations and nearby candidate genes. For example, a study from Sivasubramanian et al. (2017) identified potential genes involved with resistance to *Alternaria* in *Arabidopsis thaliana* based on their novel QTL map. The QTL mapping identified two disease-related QTL and then aligned the flanking markers to find candidate genes within those regions. In this study, this basic approach was demonstrated successfully but recall that limited genomic resources in chokecherry required the use of peach and sweet cherry reference genomes. Nevertheless, it can be expected that X-disease-related candidate genes identified in Supplementary Tables 4, 5 will have true chokecherry orthologs that can be studied in more detail.

The paucity of genetic information on chokecherry is a big obstacle for studying the mechanisms of its genetic resistance to X-disease. The chokecherry genetic linkage map constructed in this study represents a high-quality framework that can be used for the elucidation of the X-disease defense and/or susceptibility factors found in woody plant species, especially in the *Prunus* genus. Biotechnology techniques such as CRISPR-Cas9 offer unprecedented opportunities for candidate gene targeting and gene transfer to important crop species like peach and sweet cherry. The present “Cho” map has been instrumental in QTL analysis and will be a reliable reference for future molecular genetics and genomic research on X-disease and other phytoplasma plant hosts.

All data are available in the paper and supplementary materials. Data of the genetic linkage map and QTL are also archived at https://www.rosaceae.org/publication_datasets (the accession number: tfGDR1034).

## Author contributions

WD and RL: conceived and designed the study. RL: performed the experiments. WD: supervised the research and guided data interpretation. RL and WD: wrote the manuscript. All authors read and approved the final manuscript.

### Conflict of interest statement

The authors declare that the research was conducted in the absence of any commercial or financial relationships that could be construed as a potential conflict of interest.
