# Supplementary material for: Mapping X-Disease Phytoplasma Resistance in Prunus virginiana
Source: Front Plant Sci. 2017 Nov 29;8:2057. doi: 10.3389/fpls.2017.02057 (PMC5712551; doi:10.3389/fpls.2017.02057)
Supplement: Supplementary file 1 [file Table1.PDF]

Supplementary Table 1. Newly designed simple sequence repeat (SSR) markers from the peach reference genome

Marker name	Marker type	Scaffold	Forward sequence	Reverse sequence	Ta[a]
**SSR8-A12**[b]	SSR	2	TGGCGTCTGAACTCCGATTG	GAACAACTTCCTTCCTCCATCT	56
SSR8-A34	SSR	2	TCTGAGCCCGTCACCTTTC	GAGAGAAACCGATTAGCACC	56
SSR8-A56	SSR	2	CAAGGCAGAAAATTGTGAGT	TTCTTATTTGTCGGTGAGG	56
SSR8-A78	SSR	2	ATTGACACTGGCACTCTTG	TCAACACCGATTCAAGCAC	56
**SSR8-A910**	SSR	2	ACGGGCTGATTTTGCCTTGG	CAACTGCGAAGTGCTTGACG	56
SSR8-A1112	SSR	2	GCCTAAGGTTATTAGGTGC	TTTCCAGTGCTGCTCTATC	56
SSR8-B12	SSR	2	GTGGAAGGAGTACAATGGATG	CCCGTCACTTTTGACTACTTG	56
SSR8-B34	SSR	2	CAAAACAGAAATTGTGAGTGG	ATCTCGGAAAGAGGTTGGT	56
SSR8-B56	SSR	2	TTTTGTGGCTCTTTACCCT	CCTTTCTAACCATCCCTCT	56
SSR8-B78	SSR	2	CCCTTGGGATACCGAATAG	AACAGTCCATCAATGCTCC	56
SSR8-B910	SSR	2	TGGAAAGTTACCCAAATGAC	TGAAAGGTTGGAACATAGAG	56
SSR8-B1112	SSR	2	TCAGGGTCTGGAGAACGGAG	TAAAATCGGGTAGGTGAGGAAC	56
SSR8-C12	SSR	2	TTTCTGGGGCAACAGTGAC	TTGAGCCTGTTTGGGATTG	56
SSR8-C34	SSR	2	CAGATGTGGTGTTTGCCGACT	GATCAACGGAAGTATAGTGGC	56
SSR8-C56	SSR	2	AAGGATTGGCAATGCGTGT	AAGACAAAGGTGAGAAAAGC	56
SSR8-C78	SSR	2	TGGTCAAAAGAAACCCAGTGG	TTTGGCACCAGTCCCGTCT	56
SSR8-C910	SSR	2	GATTTCAACCCGATTCTAC	GATAAATCCCACTACCCAT	56
SSR8-C1112	SSR	2	TCCAATCTCACAAAATCAGG	ACTTACTGAATCACCTTTGC	56
**SSR8-D12**	SSR	2	GTGTGGGCAAGAACTTATCAT	CCATTCCCAAAGTCACCGAT	56
**SSR8-D34**	SSR	4	ACTGCCCGCATTCTTGGTT	CTCTTCAGGCTTGACATACT	56
**SSR8-D56**	SSR	4	AAGCAGCATCTTCCTCTTC	TCAATACCGTCAAGATAACC	56
**SSR8-D78**	SSR	4	GTGATGATTGAATCTGCCAT	GACAAAGCTAATTCCACATC	56
SSR8-D910	SSR	4	ATTGCTGGGAAATCTGGAAG	ATCAAGCCAAACGGCAGAG	56
**SSR8-D1112**	SSR	4	TGTTGTACATGACCGTTAGC	CATTTTCACCAAGGCTTTACC	56
**SSR8-E12**	SSR	4	AGTCAGTGAGCCAGCCTATG	GGGAATGGGAACAGGAAACG	56
**SSR8-E34**	SSR	4	GAGATAAGGGCATTTTGGTCAT	CCCAACCATCAGCTAGAGC	56
SSR8-E56	SSR	4	CGCAAGGTTCATATTCTTC	CACCATCTGTCCAAATCAT	56
**SSR8-E78**	SSR	4	GCTTTGCCTTGTAGACGCTT	GTGGAGCATAAGCATACCTC	56
SSR8-E910	SSR	4	TGTTCTCATCCAAAGCAGC	TTTTCTTGAAGCACCGTGT	56
**SSR8-E1112**	SSR	4	ATGCCTGAATGAATGCTCT	AGTGGGAGATTGAGTTTGT	56
SSR8-F12	SSR	4	GCCTTGTAGACACTTCCCTG	TGTGGAACATAAGCGAACCTC	56
SSR8-F34	SSR	4	AGAACCTTGAACGATTGAC	TGTCTGGATCTTTGTTTGG	56
SSR8-F56	SSR	4	TGGGAGGTAATTTGGTGAC	AAATGGGAATCCTTGGTGT	56
**SSR8-F78**	SSR	4	GTTGTTGTTCTCATCCGAAAC	CTACCTTTGTTGTCCTCTGC	56
**SSR8-F910**	SSR	4	GTGTAGGAGTAGCCCTGGTT	AGAGGCAAAAGAGGAACAGTG	56
SSR8-F1112	SSR	4	ATTAGAGTTTAGGGTCGGTT	GCATTCCACCAGTACAACT	56
**SSR8-G12**	SSR	4	GTGACCGTTGGAGGCTGTAT	TACACCCATTTTGCTCACCC	56
**SSR8-G34**	SSR	4	CAGTGATACCTGCTACGAT	CTATCTGCTCCGATTCCTC	56
**SSR8-G56**	SSR	4	CTTTTGAAAGCAGACAGATC	GCCAGTCTAACCTTCTCAG	56
**SSR8-G78**	SSR	6	TTGCTAACAGTGGCACGCTC	AGAAAGAAGCAGTCGTCAAGC	56
SSR8-G910	SSR	6	CTTGGCAACAAAGTAAGAAC	CACTTAGAGCAATACCACTT	56
SSR8-G1112	SSR	6	CTTCTCGGGTGATGTGGTC	CGGTTGGTTGGTCAAGACAT	56
SSR8-H12	SSR	6	AACTCACAAGTTCCCAACC	GATTAGGTTTGGCAACTGAG	56
SSR8-H34	SSR	6	TTTGCTAGGTGTCCGTCTC	CGTTGATTGTAACTTCTTCG	56
SSR8-H56	SSR	6	CCTTTAGATGGGTAGTTTGC	GCACCCGTTCAAGAATCAC	56
SSR8-H78	SSR	6	ATTGCTTACTTCCCTGGTT	AGCGAGGCTTGTGATTGG	56
**SSR8-H910**	SSR	6	TGGAAAGGGTATAGCTGTG	CAAGATGACAACTGAGGCT	56
**SSR8-H1112**	SSR	6	GGTGTACGCTTCCACTATG	CGGATTATCGGACAAAGTG	56

[a] Annealing temperature (°C) for polymerase chain reaction (PCR)

[b] Bolded marker names indicate they are polymorphic in chokecherry

Supplementary Table 2. Simple sequence repeat (SSR) markers from Dettori et al. (2015) and Zhang et al. (2014) that were successfully anchored to the new chokecherry genetic map 'Cho'

Marker name	Marker type	Source species	Forward sequence	Reverse sequence	Ta[a]
RPPG1-017	SSR	Peach	GCTCATCAAAACTCTCAACCA	CCCTTTCTTCAATCCCATC	56
RPPG1-023	SSR	Peach	GGCCTTTGTTTTCTTTCCTT	GGATTTCAGTTGACCCATTT	56
RPPG1-025	SSR	Peach	GATTTGATTCCTGTGGCATT	TGGGCATTCTTTTTCTCTTC	56
RPPG1-026	SSR	Peach	CTTCTGGCACTCTTCCATTT	GTTCCCAAGTTTTCCTCTCA	51
RPPG1-029	SSR	Peach	TCACTCCAGCATTTGAACC	AGCACTGAAAACACCACAGA	56
RPPG1-041	SSR	Peach	TGTTGTAATGGATGGTGTCTTC	CTTGGTCTTGGTTTCATTCA	56
RPPG2-007	SSR	Peach	GCATCAGAAGTCCCAATCA	GCGGTGGTGTGAAACTAAA	51
RPPG2-019	SSR	Peach	TTACGTGCTTTTCCCATGA	CGCCTTATCCCCTGACTAT	49
RPPG3-030	SSR	Peach	AAACTGCCCAAAACAAAGAC	GCAACCAACAAAGATGACAA	56
RPPG3-031	SSR	Peach	AGCGGAGAGAGAATGAGATG	GCAACAATACGAACAGCAAG	58
RPPG3-038	SSR	Peach	GTTTCCCATCCCATACCTC	CAACACAAGAAGCAAGCAAG	53
RPPG3-039	SSR	Peach	CAACACGTTATTGCCCATT	GTGAGCCACATTTACTATTGAGAG	49
RPPG3-041	SSR	Peach	TGCCATTCAACAACAAACAC	TCAAGGGAACAGGGATGA	54
RPPG4-074	SSR	Peach	AGTGGCTGTTCTGGTTTGAG	GTTTGGGGTTTGGAGAGAG	58
RPPG4-076	SSR	Peach	TGCCAACTATGCTCCTATTTAC	GGATTTGAATTGCCGAACT	54
RPPG4-097	SSR	Peach	GGCATGTGAAAGCAAAAGT	CTTCCTGAAAACCCCATTC	49
RPPG5-008	SSR	Peach	CCTGAATGGCTCTCTCTTTC	TGTTGGTGGGACTAATGATG	58
RPPG5-020	SSR	Peach	CAAGAATTTGGCTTGGAACT	GTGTATCATGGACAGCTTGC	56
RPPG5-024	SSR	Peach	TTAGAAAACGGGACAAGCAC	CAACGACACCATTGAAAACT	56
RPPG6-009	SSR	Peach	GGGCTTGGCTGATAAAATAA	TGGTAAAATAGAAGAGCGAGAAG	53
RPPG6-010	SSR	Peach	ACTTGACGTAGAGAGCATACCTAA	ATTATGGGCAGAAATGGTTG	51
RPPG6-014	SSR	Peach	ACCCAATACACAAGATTGACC	CTTTGGAAGCAGGGATTAGA	53
RPPG6-018	SSR	Peach	TCTGCTATCTGTTTGGTGGA	GACTACAGTGGGGGATGAAC	58
RPPG6-024	SSR	Peach	CTTGGAGATTGGGGCATA	CACAAGATGGACTAGGCAAA	49
RPPG6-025	SSR	Peach	GATAAAAGGGTAGGTAGGTCCA	AGTCCCATGTGCTTGTTTCT	53
RPPG6-030	SSR	Peach	GATGACACCGAGTTTCGATT	CAGATCGGGTTTACGCTACT	58
RPPG6-033	SSR	Peach	CATTATCAAACCACGACCAA	AAAGCTCAACAGCGACTTCT	56
RPPG6-036	SSR	Peach	GCTATTTTCTCCACCAGCTC	GCCATAGTTGACTGCATTGAT	55
RPPG6-038	SSR	Peach	GCATAGGGTGTGTTCCTCA	CCAGTGACATCTAGCCCATT	53
RPPG7-018	SSR	Peach	TTGTCATCAGGTCGTTCATC	TCCTCCCACTCTGTATTTGG	58
RPPG7-020	SSR	Peach	GATCCAACTTCCACCACACT	CAGGGCACCATCTCTTAAAC	55
RPPG7-023	SSR	Peach	TTTAGCCATTTACCCATTTTG	CATTCCTGTTCCCTTTTTGT	56
RPPG7-026	SSR	Peach	TTTGGTGAGTGGGCTCTATT	CTATCGTTCGCTGGTCTTCT	53
RPPG7-029	SSR	Peach	CGAAGTGGAAACAGAAGATGA	GAGGTTGAAGACGGAAGATG	55
RPPG7-032	SSR	Peach	AAGGGAGGAGGATTGTGAA	TGGTAGACGGGTAGATGTTG	53
RPPG8-007	SSR	Peach	ACCACCACCTCTTCCAATC	ACCTCAAAGTGTCCCAGAAA	53
RPPG8-011	SSR	Peach	GCTTCTTCTTTGCTTGGAGT	CCGTTCATCATCTACCTTCC	53
RPPG8-014	SSR	Peach	ACTTGAATGGGCTAAAACGA	GAGAAGAAAAGAGCGTGGAG	56
RPPG8-017	SSR	Peach	AAACTATGCCTTGCTTGAGAAC	GCGGCGTTTCTTTCCTTT	53
RPPG8-020	SSR	Peach	CTGATCTGACAAAAGCACCA	TGAAGGCAACAAGAACGTAG	58
RPPG8-030	SSR	Peach	GCAAGTCAAACCACACAAGA	TGAAAGTGAAACCAACGAGA	56
RPPG8-031	SSR	Peach	ATCATGTCCTTTGGGCTCT	GGGCAAATCGAAGTTGTG	54
NAUpy_E603	SSR	Pear	GAAAGTCCTTTTATCTAATTGGAATCCT	CAGGGCAAAGCTTTCTCTTATTTT	58

[a] Annealing temperature (°C) for polymerase chain reaction (PCR)

Supplementary Table 3. Pear retrotransposon-based long terminal repeat (LTR) markers that produced polymorphisms in chokecherry

Marker name	Marker type[a]	Source species	Forward sequence	Reverse sequence	Ta[b]
PbrRE1_2	IRAP	Pear	TCAAACCAAACAATGTGGTCA		57
PbrRE1_4	IRAP	Pear	TGATTGGAAGAATGCGATCTA		57
PbrRE2_2	IRAP	Pear	ACCGTGATGGAATGGTAAGC		57
PbrRE4_1	IRAP	Pear	GATAATTGATCCGGCCCATA		57
PbrRE5_1	IRAP	Pear	AAATACCCAAGTCCCCAAGC		57
PbrRE6_1	IRAP	Pear	AAGGAATATGGTTGGAGTCCTTC		57
PbrRE7_1	IRAP	Pear	TGTTGGAGAGTTGACCTTTTG		57
PbrRE8_1	IRAP	Pear	GCATGGGCAAAGTTTTCAAT		57

[a] Marker type - IRAP: inter-retrotransposon amplified polymorphism

[b] Annealing temperature (°C) for polymerase chain reaction (PCR)
