# Supplementary material for: Mapping X-Disease Phytoplasma Resistance in Prunus virginiana
Source: Front Plant Sci. 2017 Nov 29;8:2057. doi: 10.3389/fpls.2017.02057 (PMC5712551; doi:10.3389/fpls.2017.02057)
Supplement: Supplementary file 2 [file Table2.PDF]

Supplemental Table 4. Peach candidate genes near the chokecherry X-disease QTL

QTL Location[a]	Sequence ID[b]	Gene Annotation[c]
Cho-15	XR_002270200.1	PREDICTED: Prunus persica putative disease resistance RPP13-like protein 1 (LOC18786197), transcript variant X8, misc_RNA
	XM_007212977.2	PREDICTED: Prunus persica codeine O-demethylase (LOC18781160), mRNA
	XM_020561826.1	PREDICTED: Prunus persica zinc finger CCCH domain-containing protein 56 (LOC18780661), mRNA
	XM_020555363.1	PREDICTED: Prunus persica zinc finger MYM-type protein 1-like (LOC109946722), mRNA
	XM_020556148.1	PREDICTED: Prunus persica ankyrin repeat-containing protein NPR4 (LOC18787795), transcript variant X1, mRNA
	XM_020570930.1	PREDICTED: Prunus persica receptor like protein 30 (LOC18766744), mRNA
	XR_002269659.1	PREDICTED: Prunus persica probable disease resistance protein At1g58602 (LOC18791659), transcript variant X7, misc_RNA
	XM_007226947.2	PREDICTED: Prunus persica probable alpha,alpha-trehalose-phosphate synthase [UDP-forming] 9 (LOC18791614), mRNA
	XM_007225197.2	PREDICTED: Prunus persica alpha,alpha-trehalose-phosphate synthase [UDP-forming] 6 (LOC18788532), mRNA
	XM_020561125.1	PREDICTED: Prunus persica cyclic nucleotide-gated ion channel 18 (LOC18781944), mRNA
	XM_007209972.2	PREDICTED: Prunus persica probable cyclic nucleotide-gated ion channel 16 (LOC18777380), mRNA
	XM_007221284.2	PREDICTED: Prunus persica zinc finger protein MAGPIE (LOC18787860), mRNA
	XM_020557305.1	PREDICTED: Prunus persica rust resistance kinase Lr10-like (LOC109947360), mRNA
	XM_020557506.1	PREDICTED: Prunus persica probable glycosyltransferase At5g25310 (LOC109947460), mRNA
	XM_020564791.1	PREDICTED: Prunus persica TMV resistance protein N (LOC18791623), transcript variant X1, mRNA
	XM_007209576.2	PREDICTED: Prunus persica growth-regulating factor 4 (LOC18776814), mRNA
	XM_007212548.2	PREDICTED: Prunus persica receptor-like protein 2 (LOC18779499), mRNA
	XM_007208576.2	PREDICTED: Prunus persica probable LRR receptor-like serine/threonine-protein kinase At1g06840 (LOC18773395), mRNA

QTL Location	Sequence ID	Gene Annotation
Cho-5	XM_020569195.1	PREDICTED: Prunus persica WAT1-related protein At1g68170 (LOC18769598), mRNA
	XM_020561269.1	PREDICTED: Prunus persica protein ROOT HAIR DEFECTIVE 3 homolog 2 (LOC18779831), mRNA
	XM_020569670.1	PREDICTED: Prunus persica bromodomain-containing factor 1 (LOC18768427), transcript variant X2, mRNA
	XM_020569622.1	PREDICTED: Prunus persica transcription factor GTE11 (LOC18767947), transcript variant X4, mRNA
	XM_020570922.1	PREDICTED: Prunus persica probable leucine-rich repeat receptor-like protein kinase At5g63930 (LOC18768418), mRNA
	XM_007200084.2	PREDICTED: Prunus persica probable aspartyl protease At4g16563 (LOC18766967), mRNA

	XM_020569549.1	PREDICTED: Prunus persica probable leucine-rich repeat receptor-like protein kinase At2g33170 (LOC18767494), mRNA
	XM_007207894.2	PREDICTED: Prunus persica probable aspartyl protease At4g16563 (LOC18775368), mRNA
	XM_020569674.1	PREDICTED: Prunus persica ankyrin repeat, bromo and BTB domain-containing protein DDB_G0293800 (LOC18767477), transcript variant X1, mRNA
	XM_020570002.1	PREDICTED: Prunus persica TMV resistance protein N (LOC18767635), transcript variant X1, mRNA
	XM_020570754.1	PREDICTED: Prunus persica GDSL esterase/lipase At4g16230 (LOC18767043), mRNA
	XM_007204416.2	PREDICTED: Prunus persica 1-aminocyclopropane-1-carboxylate oxidase homolog 4 (LOC18770035), mRNA
	XM_020568637.1	PREDICTED: Prunus persica beta-fructofuranosidase, insoluble isoenzyme 1 (LOC18771178), mRNA

QTL Location	Sequence ID	Gene Annotation
Cho-4	XM_007213648.2	PREDICTED: Prunus persica phytosulfokine receptor 2 (LOC18779076), mRNA
	XM_020563402.1	PREDICTED: Prunus persica probable WRKY transcription factor 15 (LOC18776508), mRNA
	XM_020568844.1	PREDICTED: Prunus persica probable disease resistance protein At4g27220 (LOC18771471), mRNA
	XM_020561265.1	PREDICTED: Prunus persica alpha,alpha-trehalose-phosphate synthase [UDP-forming] 1 (LOC18780844), transcript variant X1, mRNA
	XM_020561640.1	PREDICTED: Prunus persica cation/H(+) antiporter 4 (LOC18780436), mRNA
	XM_020561950.1	PREDICTED: Prunus persica transcription factor GTE12 (LOC18778974), mRNA
	XM_007214050.2	PREDICTED: Prunus persica pollen-specific leucine-rich repeat extensin-like protein 1 (LOC18780766), mRNA
	XM_007213578.2	PREDICTED: Prunus persica subtilisin-like protease SBT5.6 (LOC18781277), mRNA
	XM_007211884.2	PREDICTED: Prunus persica bidirectional sugar transporter SWEET2a (LOC18780140), mRNA
	XM_007213404.2	PREDICTED: Prunus persica PLASMODESMATA CALLOSE-BINDING PROTEIN 1 (LOC18778757), mRNA
	XM_007213642.2	PREDICTED: Prunus persica probable leucine-rich repeat receptor-like serine/threonine-protein kinase At3g14840 (LOC18781279), mRNA
	XM_020562774.1	PREDICTED: Prunus persica probable LRR receptor-like serine/threonine-protein kinase RFK1 (LOC18780480), transcript variant X1, mRNA
	XM_007211718.2	PREDICTED: Prunus persica xyloglucan endotransglucosylase/hydrolase 2 (LOC18781193), mRNA
	XM_020562770.1	PREDICTED: Prunus persica probable LRR receptor-like serine/threonine-protein kinase At1g07650 (LOC18781287), mRNA
	XM_020561779.1	PREDICTED: Prunus persica serine/threonine-protein kinase TNNI3K (LOC18778874), mRNA
	XM_007214496.2	PREDICTED: Prunus persica probable leucine-rich repeat receptor-like serine/threonine-protein kinase At3g14840 (LOC18781425), mRNA
	XM_007211409.2	PREDICTED: Prunus persica GDSL esterase/lipase At5g45670 (LOC18779850), mRNA

XM_007213616.2	PREDICTED: Prunus persica probable LRR receptor-like serine/threonine-protein kinase At1g07650 (LOC18780447), mRNA
XM_007213038.2	PREDICTED: Prunus persica GDSL esterase/lipase At1g29670 (LOC18780199), mRNA
XM_007211919.2	PREDICTED: Prunus persica cold-regulated 413 inner membrane protein 2, chloroplastic (LOC18779742), mRNA
XR_002271509.1	PREDICTED: Prunus persica uncharacterized LOC109949017 (LOC109949017), transcript variant X1, ncRNA
XM_007212913.2	PREDICTED: Prunus persica MDIS1-interacting receptor like kinase 2 (LOC18779095), mRNA
XM_007211489.2	PREDICTED: Prunus persica organic cation/carnitine transporter 3 (LOC18780040), mRNA
XR_002271510.1	PREDICTED: Prunus persica uncharacterized LOC109949017 (LOC109949017), transcript variant X2, ncRNA
XM_007211889.2	PREDICTED: Prunus persica metacaspase-4 (LOC18780792), mRNA
XM_020561628.1	PREDICTED: Prunus persica la-related protein 1 (LOC18779758), mRNA
XM_007211401.2	PREDICTED: Prunus persica myb family transcription factor APL (LOC18778208), mRNA
XM_020562832.1	PREDICTED: Prunus persica peroxidase 7 (LOC18779847), mRNA
XM_007212929.2	PREDICTED: Prunus persica proline-rich receptor-like protein kinase PERK2 (LOC18778619), mRNA
XM_020562197.1	PREDICTED: Prunus persica myb-related protein 330 (LOC18780806), mRNA
XM_007211343.2	PREDICTED: Prunus persica uncharacterized LOC18779610 (LOC18779610), mRNA
XM_020562090.1	PREDICTED: Prunus persica probable LRR receptor-like serine/threonine-protein kinase At3g47570 (LOC18781198), mRNA
XM_007212798.2	PREDICTED: Prunus persica organic cation/carnitine transporter 3 (LOC18781497), mRNA
XM_007212736.2	PREDICTED: Prunus persica zinc transporter 1 (LOC18780316), mRNA
XM_020563110.1	PREDICTED: Prunus persica protein FORGETTER 1 (LOC18778728), transcript variant X1, mRNA
XM_007213035.2	PREDICTED: Prunus persica 18.1 kDa class I heat shock protein (LOC18778516), transcript variant X1, mRNA
XM_007213680.2	PREDICTED: Prunus persica succinate-semialdehyde dehydrogenase, mitochondrial (LOC18778265), transcript variant X1, mRNA
XM_007214013.2	PREDICTED: Prunus persica NAC domain-containing protein 86 (LOC18778986), mRNA
XM_007214536.2	PREDICTED: Prunus persica (E,E)-alpha-farnesene synthase (LOC18779527), mRNA

[a] The flanking markers of the QTL position were aligned to the peach genome and BLAST was used to find gene models related to disease, stress, and regulation

[b] Sequence IDs correspond to the peach reference genome

[c] Gene annotations were given by NCBI BLAST and analyzed for gene ontologies via UniProtKB

Supplemental Table 5. Sweet cherry candidate genes near the chokecherry X-disease QTL

QTL Location[a]	Sequence ID[b]	Gene Annotation[c]
Cho-15	XM_021959597.1	PREDICTED: Prunus avium protein ACCELERATED CELL DEATH 6-like (LOC110757860), mRNA
	XM_021964886.1	PREDICTED: Prunus avium zinc finger MYM-type protein 1-like (LOC110762283), mRNA
	XM_021957625.1	PREDICTED: Prunus avium ubiquitin carboxyl-terminal hydrolase 24-like (LOC110756222), mRNA
	XM_021959602.1	PREDICTED: Prunus avium transient receptor potential cation channel subfamily A member 1-like (LOC110757864), transcript variant X2, mRNA
	XM_021945270.1	PREDICTED: Prunus avium receptor-like protein 12 (LOC110745196), partial mRNA
	XM_021978735.1	PREDICTED: Prunus avium probable leucine-rich repeat receptor-like serine/threonine-protein kinase At3g14840 (LOC110774194), mRNA
	XM_021975368.1	PREDICTED: Prunus avium WD repeat-containing protein 91-like (LOC110771124), transcript variant X1, mRNA
	XM_021952048.1	PREDICTED: Prunus avium probable serine/threonine-protein kinase At4g35230 (LOC110751556), mRNA
	XM_021969702.1	PREDICTED: Prunus avium probable alpha,alpha-trehalose-phosphate synthase [UDP-forming] 9 (LOC110766383), mRNA
	XM_021969700.1	PREDICTED: Prunus avium cyclic nucleotide-gated ion channel 17-like (LOC110766382), mRNA
	XM_021958043.1	PREDICTED: Prunus avium UDP-glycosyltransferase 76F1-like (LOC110756600), mRNA
	XM_021974663.1	PREDICTED: Prunus avium proline-rich receptor-like protein kinase PERK4 (LOC110770511), mRNA
	XM_021968926.1	PREDICTED: Prunus avium probable sodium/metabolite cotransporter BASS2, chloroplastic (LOC110765720), mRNA
	XM_021967677.1	PREDICTED: Prunus avium pectinesterase inhibitor 4-like (LOC110764666), mRNA
	XM_021968722.1	PREDICTED: Prunus avium cytochrome P450 CYP749A22-like (LOC110765579), mRNA
	XM_021948934.1	PREDICTED: Prunus avium CASP-like protein 5B3 (LOC110748954), mRNA
	XM_021965051.1	PREDICTED: Prunus avium disease resistance RPP13-like protein 4 (LOC110762414), transcript variant X1, mRNA
	XM_021968151.1	PREDICTED: Prunus avium disease resistance protein At4g27190-like (LOC110765098), partial mRNA
	XM_021963313.1	PREDICTED: Prunus avium putative calcium-transporting ATPase 13, plasma membrane-type (LOC110760953), mRNA
	XM_021975000.1	PREDICTED: Prunus avium protein STRICTOSIDINE SYNTHASE-LIKE 5-like (LOC110770779), mRNA
	XM_021961384.1	PREDICTED: Prunus avium zinc finger protein NUTCRACKER (LOC110759330), mRNA
	XM_021977473.1	PREDICTED: Prunus avium isoflavone reductase homolog (LOC110772980), mRNA
	XM_021953769.1	PREDICTED: Prunus avium methylesterase 3-like (LOC110752981), transcript variant X2, mRNA
QTL Location	Sequence ID	Annotation

Cho-5	XM_021944225.1	PREDICTED: Prunus avium LRR receptor-like serine/threonine-protein kinase RCH1 (LOC110744258), mRNA
	XM_021944229.1	PREDICTED: Prunus avium LRR receptor-like serine/threonine-protein kinase GSO1 (LOC110744262), mRNA
	XM_021944228.1	PREDICTED: Prunus avium probable leucine-rich repeat receptor-like protein kinase At2g33170 (LOC110744261), mRNA
	XM_021957644.1	PREDICTED: Prunus avium LRR receptor-like serine/threonine-protein kinase RCH1 (LOC110756243), mRNA
	XM_021947233.1	PREDICTED: Prunus avium LRR receptor-like serine/threonine-protein kinase RCH1 (LOC110747016), partial mRNA
	XM_021944283.1	PREDICTED: Prunus avium transcription factor GTE8-like (LOC110744310), transcript variant X1, mRNA
	XM_021949559.1	PREDICTED: Prunus avium probable aspartyl protease At4g16563 (LOC110749445), mRNA
	XM_021946297.1	PREDICTED: Prunus avium transcription factor GTE11-like (LOC110746088), transcript variant X1, mRNA
	XM_021977717.1	PREDICTED: Prunus avium LRR receptor-like serine/threonine-protein kinase ERL2 (LOC110773214), mRNA
	XM_021977723.1	PREDICTED: Prunus avium GDSL esterase/lipase At4g16230-like (LOC110773222), mRNA
	XM_021977715.1	PREDICTED: Prunus avium LRR receptor-like serine/threonine-protein kinase (LOC110773212), mRNA
	XM_021975210.1	PREDICTED: Prunus avium protein ACCELERATED CELL DEATH 6-like (LOC110770989), transcript variant X1, mRNA
	XM_021969598.1	PREDICTED: Prunus avium transcription factor MYB26-like (LOC110766298), mRNA
	XM_021957423.1	PREDICTED: Prunus avium rust resistance kinase Lr10 (LOC110756046), mRNA
	XM_021975489.1	PREDICTED: Prunus avium TMV resistance protein N-like (LOC110771225), mRNA
	XM_021965051.1	PREDICTED: Prunus avium disease resistance RPP13-like protein 4 (LOC110762414), transcript variant X1, mRNA
	XM_021968151.1	PREDICTED: Prunus avium disease resistance protein At4g27190-like (LOC110765098), partial mRNA
	XR_002527409.1	PREDICTED: Prunus avium probable transcription factor KAN2 (LOC110761587), transcript variant X5, misc_RNA
	XM_021977691.1	PREDICTED: Prunus avium skeletal aspartic acid-rich protein 1-like (LOC110773187), mRNA
	XM_021963930.1	PREDICTED: Prunus avium protein ROOT HAIR DEFECTIVE 3 homolog 2-like (LOC110761457), transcript variant X2, mRNA
	XM_021963462.1	PREDICTED: Prunus avium 1-aminocyclopropane-1-carboxylate oxidase homolog 4-like (LOC110761078), mRNA
	XM_021963967.1	PREDICTED: Prunus avium beta-fructofuranosidase, insoluble isoenzyme 1-like (LOC110761484), mRNA
	XM_021963518.1	PREDICTED: Prunus avium WD repeat-containing protein LWD1-like (LOC110761118), mRNA
	XM_021964068.1	PREDICTED: Prunus avium NAC domain-containing protein 90-like (LOC110761576), mRNA
	XM_021975210.1	PREDICTED: Prunus avium protein ACCELERATED CELL DEATH 6-like (LOC110770989), transcript variant X1, mRNA
	XM_021962810.1	PREDICTED: Prunus avium cytochrome P450 71B36-like (LOC110760531), mRNA

QTL Location	Sequence ID	Annotation
	XM_021975000.1	PREDICTED: Prunus avium protein STRICTOSIDINE SYNTHASE-LIKE 5-like (LOC110770779), mRNA
QTL Location	Sequence ID	Annotation
Cho-4	XM_021973103.1	PREDICTED: Prunus avium phytosulfokine receptor 2 (LOC110769174), mRNA
	XM_021966313.1	PREDICTED: Prunus avium transcription factor ORG2-like (LOC110763517), mRNA
	XM_021952777.1	PREDICTED: Prunus avium MDIS1-interacting receptor like kinase 2-like (LOC110752174), mRNA
	XM_021962551.1	PREDICTED: Prunus avium organic cation/carnitine transporter 3-like (LOC110760303), mRNA
	XM_021974697.1	PREDICTED: Prunus avium probable leucine-rich repeat receptor-like protein kinase At1g35710 (LOC110770548), mRNA
	XM_021962555.1	PREDICTED: Prunus avium serine/threonine-protein kinase LMTK3 (LOC110760307), mRNA
	XM_021957190.1	PREDICTED: Prunus avium leucine-rich repeat extensin-like protein 3 (LOC110755887), mRNA
	XM_021962569.1	PREDICTED: Prunus avium organic cation/carnitine transporter 3-like (LOC110760320), mRNA
	XM_021957186.1	PREDICTED: Prunus avium zinc transporter 1-like (LOC110755884), mRNA
	XM_021978343.1	PREDICTED: Prunus avium metal tolerance protein 10-like (LOC110773822), mRNA
	XM_021974691.1	PREDICTED: Prunus avium ent-kaur-16-ene synthase, chloroplastic-like (LOC110770543), mRNA
	XM_021962548.1	PREDICTED: Prunus avium (E,E)-alpha-farnesene synthase-like (LOC110760301), mRNA
	XM_021957170.1	PREDICTED: Prunus avium programmed cell death protein 2-like (LOC110755869), transcript variant X1, mRNA
	XM_021957137.1	PREDICTED: Prunus avium receptor kinase-like protein Xa21 (LOC110755851), mRNA
	XM_021969917.1	PREDICTED: Prunus avium alpha,alpha-trehalose-phosphate synthase [UDP-forming] 1-like (LOC110766572), mRNA
	XM_021969993.1	PREDICTED: Prunus avium two-pore potassium channel 3-like (LOC110766628), transcript variant X1, mRNA
	XM_021969947.1	PREDICTED: Prunus avium cation/H(+) antiporter 4-like (LOC110766595), mRNA
	XM_021945059.1	PREDICTED: Prunus avium protein NRT1/ PTR FAMILY 4.5-like (LOC110745005), mRNA
	XM_021969926.1	PREDICTED: Prunus avium transcription factor GTE12-like (LOC110766577), mRNA
	XM_021945057.1	PREDICTED: Prunus avium MACPF domain-containing protein CAD1 (LOC110745004), mRNA
	XM_021969919.1	PREDICTED: Prunus avium zinc transport protein ZntB-like (LOC110766574), transcript variant X1, mRNA
	XM_021969950.1	PREDICTED: Prunus avium subtilisin-like protease SBT5.6 (LOC110766597), mRNA
	XR_002526617.1	PREDICTED: Prunus avium probable LRR receptor-like serine/threonine-protein kinase At1g07650 (LOC110756118), misc_RNA
	XM_021969916.1	PREDICTED: Prunus avium PLASMODESMATA CALLOSE-BINDING PROTEIN 1-like (LOC110766570), mRNA

XM_021969996.1	PREDICTED: Prunus avium bidirectional sugar transporter SWEET2a-like (LOC110766630), mRNA
XM_021945060.1	PREDICTED: Prunus avium oil body-associated protein 2A-like (LOC110745006), mRNA
XM_021969976.1	PREDICTED: Prunus avium serine/threonine-protein kinase TNNI3K (LOC110766613), mRNA
XM_021969903.1	PREDICTED: Prunus avium xyloglucan endotransglucosylase/hydrolase 2-like (LOC110766561), mRNA
XM_021945050.1	PREDICTED: Prunus avium serine/threonine-protein kinase TNNI3K-like (LOC110745000), mRNA
XM_021945391.1	PREDICTED: Prunus avium probable LRR receptor-like serine/threonine-protein kinase At1g07650 (LOC110745317), mRNA
XM_021969992.1	PREDICTED: Prunus avium cold-regulated 413 inner membrane protein 2, chloroplastic-like (LOC110766626), mRNA
XM_021969907.1	PREDICTED: Prunus avium GDSL esterase/lipase At1g29660-like (LOC110766564), mRNA
XM_021967634.1	PREDICTED: Prunus avium probable LRR receptor-like serine/threonine-protein kinase At1g07650 (LOC110764626), mRNA
XM_021969991.1	PREDICTED: Prunus avium eukaryotic translation initiation factor 4E-1 (LOC110766625), mRNA
XM_021945551.1	PREDICTED: Prunus avium probable LRR receptor-like serine/threonine-protein kinase At1g07650 (LOC110745462), mRNA
XM_021957476.1	PREDICTED: Prunus avium probable leucine-rich repeat receptor-like serine/threonine-protein kinase At3g14840 (LOC110756094), partial mRNA
XM_021969909.1	PREDICTED: Prunus avium GDSL esterase/lipase At5g45670-like (LOC110766566), mRNA

[a] The flanking markers of the QTL position were aligned to the sweet cherry genome on the GDR website and BLAST was used to find gene models related to disease, stress, and regulation

[b] Sequence IDs correspond to the peach reference genome

[c] Gene annotations were given by NCBI BLAST and analyzed for gene ontologies via UniProtKB
