# Supplementary figures and images for: Mapping X-Disease Phytoplasma Resistance in Prunus virginiana
Source: Front Plant Sci. 2017 Nov 29;8:2057. doi: 10.3389/fpls.2017.02057 (PMC5712551; doi:10.3389/fpls.2017.02057)

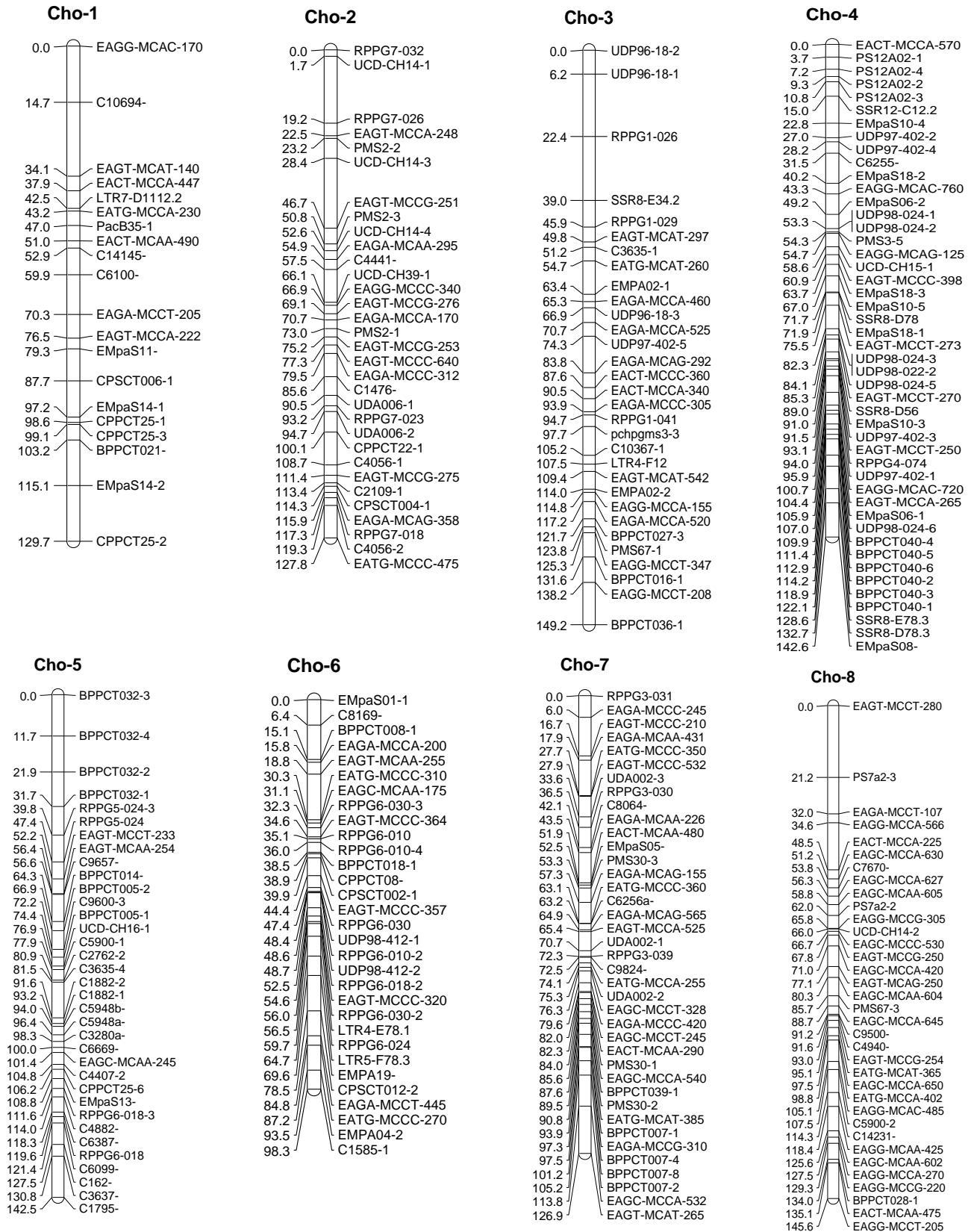

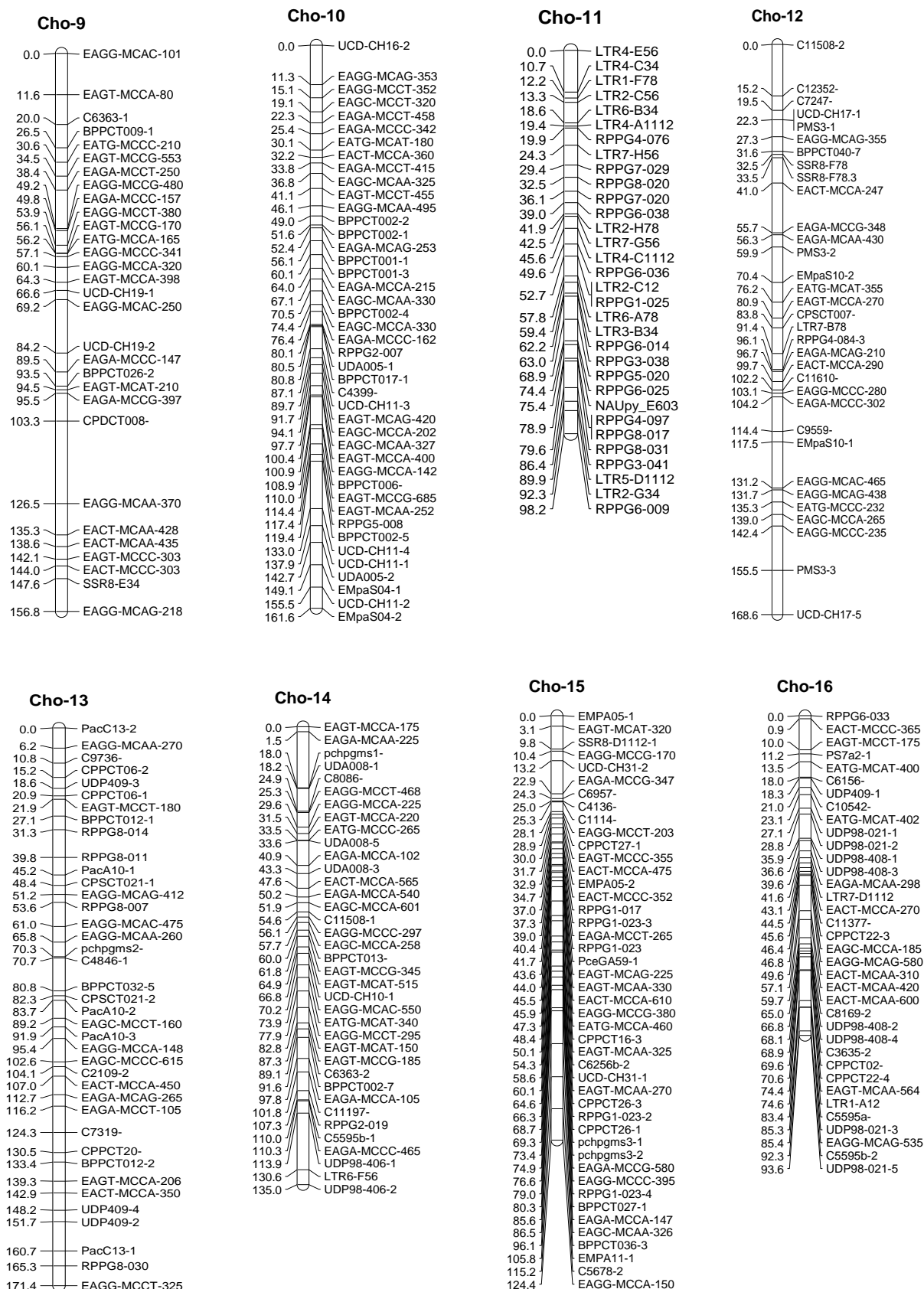

SupplementaryFigure 1. Linkage groups 1-16 of the new chokecherry genetic map 'Cho'

Supplement: Supplementary file 3 [file Image1.PDF]
