# Supplementary material for: Mapping X-Disease Phytoplasma Resistance in Prunus virginiana
Source: Front Plant Sci. 2017 Nov 29;8:2057. doi: 10.3389/fpls.2017.02057 (PMC5712551; doi:10.3389/fpls.2017.02057)
Supplement: Supplementary file 4 [file Image2.PDF]

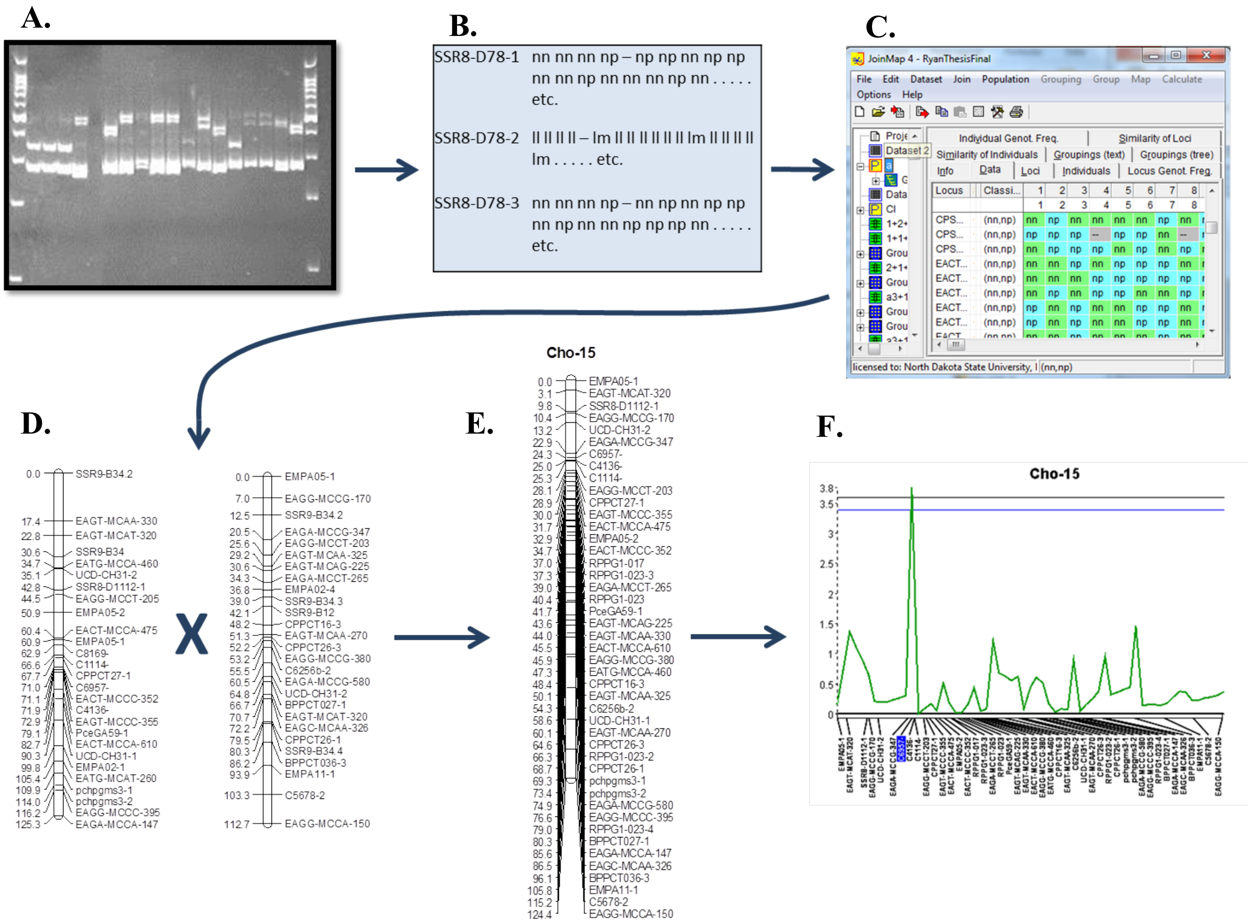

Supplementary Figure 2. A flowchart summarizing the steps taken to create a genetic linkage map and identify quantitative trait loci (QTL) in chokecherry. **A.** Gel electrophoresis image of amplified alleles from a single PCR-based molecular marker (i.e. SSR) **B.** The amplicons presented in the gel image are recorded individually as 'single dose' alleles according to the coding requirements of the utilized software package **C.** A collection of all the genotyping data is uploaded to a software package (i.e. JoinMap) and tests are run (i.e. segregation distortion) to eliminate molecular markers from the genetic mapping process. Genetic mapping is conducted within the software program to develop separate parental linkage groups **D.** Parental linkage groups are compared via MapChart to identify shared genetic loci for integration **E.** Homologous parental linkage groups as indicated by shared loci are combined to produce an integrated consensus map **F.** Progeny genotype data within each linkage group is associated with its corresponding phenotypic (i.e. disease resistance) score and analyzed via composite interval mapping and permutation within a QTL analysis software program (i.e. QGene)
